# UAV-OBB: An aerial urban vehicle dataset with oriented bounding boxes for remote sensing object detection in smart cities

**DOI:** 10.1016/j.dib.2026.112710

**Published:** 2026-03-25

**Authors:** Israr Ahmad, Shang Fengjun, Kiran Bibi, Muhammad Salman Pathan

**Affiliations:** a School of Computer Science and Technology, Chongqing University of Posts and Telecommunications, Chongqing, China; b Key Laboratory of Computer Network and Communication Technology, Chongqing, China; c Beijing Language and Culture University, Beijing, China; d ADAPT SFI Research Centre, School of Computing, Dublin City University, Ireland

**Keywords:** Overhead imagery, Nadir-view acquisition, Rotated object annotation, YOLOv8-OBB labels, Multi-scale targets, Occlusion and truncation, Aerial traffic surveillance

## Abstract

Urban smart city traffic management increasingly relies on UAV-based sensing, yet many widely used drone datasets annotate vehicles with axis-aligned bounding boxes that include unnecessary background and do not encode vehicle orientation. We present UAV-OBB, an aerial urban vehicle dataset with oriented bounding boxes (OBBs), designed for rotation-aware computer vision object detection and traffic monitoring from predominantly nadir-view UAV imagery. UAV-OBB contains 1617 RGB images at 1920 × 1080 resolution captured over roads in Chongqing and Wuhan (China), together with OBB Nannotations in YOLOv8-OBB label format and supplementary MP4 evaluation videos. The dataset provides 46,807 oriented annotations across six vehicle classes: bike, bus, car, other_vehicle, taxi, and truck, and is split into 1383 training images, 218 validation images, and 16 test images. Data were collected at 75 to 108 m altitude under diverse real-world conditions, including morning, midday, evening, night, rain, and mist or light fog, with both wide field-of-view and zoom settings to introduce strong scale variation. All instances were manually annotated using rotation-capable tools and double-checked for consistency, and occluded and truncated vehicles were included when the majority of the object was visible. To support practical smart city evaluation beyond static mAP, UAV-OBB also includes a short video clip with sparsely annotated reference frames and a longer unannotated sequence for qualitative assessment of temporal stability and deployment behaviour. UAV-OBB provides a realistic benchmark for rotation-aware detection, tracking, counting, and traffic flow analysis in urban UAV surveillance scenarios.

Specifications Table

**Table:** 

Subject	Computer Sciences
Specific subject area	UAV-based traffic object detection in urban environments with oriented bounding boxes.
Type of data	RGB annotated images (JPEG), annotations (text files in YOLOv8 OBB label format), and video (MP4).
Data collection	DJI Mavic 3 UAV captured top-down images at 75–108 m altitude over city roads; frames extracted from 4 K aerial video (3840 × 2160, 30 fps) for manual annotation of vehicles.
Data source location	City/Region: Chongqing City, and Wuhan (Hubei), P.R. China.
Data format	Raw JPEG images (1920 × 1080); annotation text files (.txt) with object class and oriented bounding box (c_x, c_y, w, h, θ) per YOLOv8-OBB conventions; MP4 video files (supplementary evaluation sequences).
Data accessibility	Ahmad, Israr; Fengjun, Shang; Bibi, Kiran; Slaman Pathan, Muhammad (2026), “UAV-OBB: An Aerial Urban Vehicle Dataset with Oriented Bounding Boxes for Remote Sensing Object Detection in Smart Cities”, Mendeley Data, V4, doi:10.17632/6snrjwcpkh.4 Repository name: Mendeley Data. Data identification number: 10.17632/6snrjwcpkh.4 Direct URL to data: https://data.mendeley.com/datasets/6snrjwcpkh/4
Related research article	None (this is the primary data descriptor; dataset not previously published in a research article).

## 1 Value of the Data

• The UAV-OBB dataset addresses a clear gap in current computer vision resources. Unlike previous UAV- or drone-based datasets, such as VisDrone [1] and UAVDT [2], which rely on axis-aligned bounding boxes that often include extra background area around the target objects and frequently feature oblique camera angles or non-urban scenes, our dataset offers nadir-view (top-down) imagery with oriented bounding box annotations tailored to urban UAV traffic monitoring. This design allows models to directly learn object orientations, enabling more precise localization than is possible with horizontal bounding boxes [3]. By focusing on overhead vehicle detection under diverse real-world conditions, UAV-OBB advances rotation-aware object detection and supports robust vehicle tracking for urban traffic surveillance. It also provides a realistic benchmark for evaluating algorithms in scenarios that closely reflect practical aerial monitoring applications, contributing to progress in aerial object detection.
• The dataset captures multi-scale objects and varied conditions, such as different times of day and weather, challenging models to detect vehicles ranging from distant tiny bikes to close-up large buses and trucks under rainy and slightly foggy conditions. Such diversity supports researchers in designing scale-aware detection algorithms and evaluating the robustness of vision models against real-world environmental variation. The presence of occluded and truncated vehicles further enables the development of occlusion-resistant object detectors from a smart city perspective and allows models to generalize better than existing aerial image benchmarks such as DOTA, which spans 18 categories but includes only two related to urban daily life traffic scenarios.
• Beyond core object detection, UAV-OBB enables: (i) Training rotation-aware detectors for dense urban traffic; oriented bounding box annotations allow models to learn vehicle heading, improving localization accuracy for turning vehicles and reducing background noise in crowded intersections. Domain adaptation research across weather conditions; the dataset's coverage of morning, midday, evening, night, rain, and mist/fog scenarios supports studies on model robustness and transfer learning under environmental distribution shifts. Human-in-the-loop evaluation using supplementary videos; the included annotated and unannotated video sequences enable qualitative assessment of temporal consistency, false-positive accumulation, and deployment behavior, complementing static image metrics with real-world monitoring insights.
• The comprehensive annotations, including oriented boxes for every vehicle class, and the supplementary video sequences add value for various research tasks. Beyond static object detection, UAV-OBB supports multi-object tracking through annotated video clips, UAV-based automatic monitoring of traffic situations or behaviors, traffic counting in specific areas, traffic density estimation, vehicle trajectory analysis, and even domain adaptation studies.
• The data is useful not only for object detection but also for researchers in computer vision, urban planning, and intelligent transportation to develop and evaluate algorithms for vehicle crowd-flow analysis and rotation-invariant detection models. The dataset’s split into train, validation, and test sets, along with additional videos, allows both quantitative evaluation and qualitative assessment of model performance. The small test set encourages external validation using the videos, simulating live traffic monitoring scenarios and supporting the development of human-in-the-loop evaluation methods for aerial detection. The availability of consistent oriented bounding boxes with ground truth means that algorithms leveraging rotation information can be directly trained and tested, which can lead to improved performance in detecting and especially in tracking vehicles at arbitrary angles in smart city contexts.

## 2 Background

Recent progress in UAV-based remote sensing has made aerial video and imagery a practical input for urban traffic observation, where vehicles may appear at arbitrary headings due to lane geometry, turning manoeuvres, and intersection layouts. However, many commonly used UAV/drone benchmarks in this area primarily provide horizontal (axis-aligned) bounding boxes, which do not explicitly represent object rotation and can introduce label-image mismatch when targets are elongated and rotated. In parallel, rotation-aware detection methods such as, rotated anchors and oriented region-based representations rely on training data that encodes orientation in a consistent annotation scheme, yet publicly available UAV traffic imagery with such labels remains limited.

Against this methodological backdrop, we compiled UAV-OBB to supply nadir-dominant urban road imagery with manually created oriented vehicle annotations suitable for modern rotation-aware detectors. The dataset was assembled from UAV video by selecting representative frames under varied capture conditions and then labelling each visible vehicle with a rotation-capable workflow, followed by verification to promote consistency. Labels are provided in a YOLO-oriented format to facilitate direct use in contemporary training pipelines and straightforward conversion to alternative representations when needed*.*

## 3 Data Description

*UAV-OBB consists of 1375 images and their corresponding annotation files, partitioned into train, validation, and test sets. The dataset is released in a YOLO-style directory layout. The root folder UAV-OBB/, the dataset configuration file (data.yaml), and supplementary MP4 evaluation videos, organized as follows:*

***UAV-OBB/***

***train/***

***images/*** 1

***labels/***

***valid/***

***images/***

***labels/***

***test/***

***images/***

***labels/***

***data.yaml*** 2

***test_videos_mp4/*** 3

All images are 1920 × 1080 pixels (8-bit RGB) in resolution and in JPEG format, captured by UAV cameras in two Chinese cities. The images predominantly show urban road scenes from a bird’s-eye view. Each image is accompanied by a text file in YOLOv8 oriented bounding box format, where each line contains an object’s class label and normalized bounding box parameters (center x, center y, width, height, angle). The angle is measured with respect to the image’s horizontal axis, allowing the bounding box to align with the vehicle’s orientation on the road. The ground sampling distance varies with altitude, but vehicles are generally clearly visible; typical drone flight altitude was about 75–108 m above ground for wide-area coverage.

***Class Annotations:*** We provide oriented bounding box annotations for six vehicle classes commonly found in urban traffic: (1) bike (including motorcycles, scooter, and sometimes bicycles), (2) bus (city buses or coaches), (3) car (private and personal automobiles), (4) other_vehicle (vehicles not in other categories, such as three-wheelers, cargo vehicles, and normal delivery vehicles), (5) taxi (taxis, marked separately due to their distinct appearance and importance in traffic analysis), and (6) truck (large trucks). **Detailed definitions, visual cues, and total counts for these six classes are summarized in** Table 2. Each object instance is annotated with a rotated rectangle aligned to the object’s orientation. We adopt the YOLOv8 Oriented Bounding Box (YOLOv8-OBB) format for labels, which encodes each object as (class_id, x_center, y_center, width, height, θ) where (x_center, y_center) is the polygon center relative to image, width and height are the bounding box dimensions, and θ is the rotation angle (in degrees) of the box with respect to the horizontal axis. Using oriented boxes allows tight encapsulation of slanting vehicles (for example, cars turning at an intersection) that axis-aligned boxes would otherwise cover inefficiently with extra background. All annotations were manually drawn and double-checked by human labelers, ensuring each vehicle’s box matches its footprint and orientation as closely as possible. Partially visible vehicles at image borders are annotated (with their box truncated by the image edge), and even severely occluded vehicles were also annotated to maintain label quality and make a real urban scene.

***Data Collection Conditions:*** The images were collected under six distinct conditions to maximize diversity:

• Morning: Early daylight hours with long shadows and warm light.
• Midday daylight: Bright conditions with overhead sun, minimal shadows.
• Evening: Dusk or low-light conditions, including scenarios just before or after sunset with artificial street lighting starting to appear.
• Night: Dark conditions with street lighting and vehicle headlights, featuring high contrast between lit roads and dark surroundings.
• Rain: Light rainfall conditions, causing reflective road surfaces and potential motion blur due to water; vehicles often have headlights on.
• Mist/Fog: Reduced visibility conditions with light fog or smog, causing lower image contrast and partially veiling distant objects.

These conditions reflect real challenges in urban traffic for all-day, night, and all-weather monitoring. By including such variety, the UAV-OBB dataset tests detection algorithms’ robustness to illumination changes and atmospheric effects that are common in city environments.

**Fig. 1:**
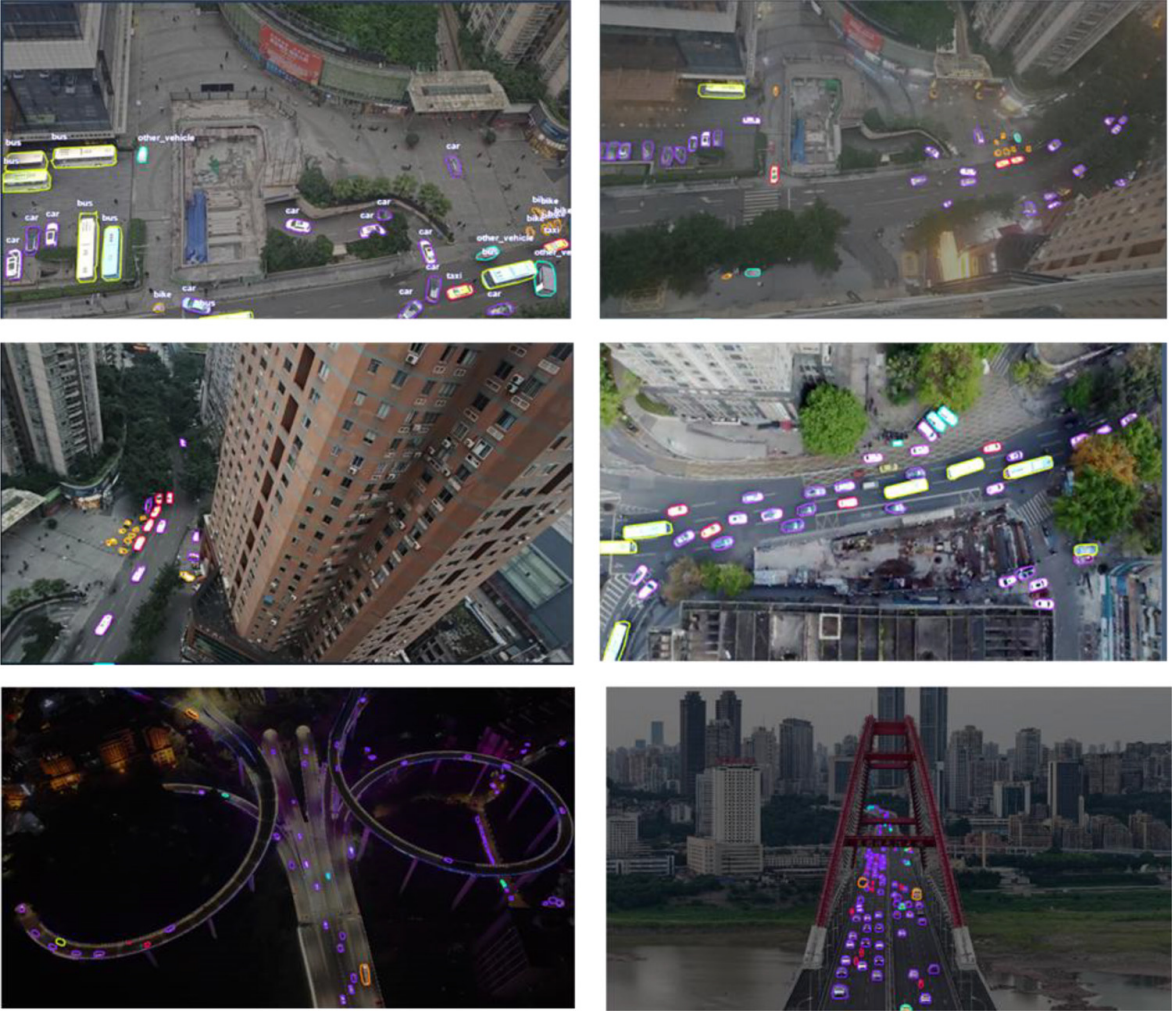
Sample imagery from the UAV-OBB dataset displaying oriented bounding box annotations for vehicles across diverse urban scenes including bridges, intersections, and highways.

***Scale Variation:*** A notable feature of UAV-OBB is the scale variance introduced deliberately through camera settings. For many scenes, we captured two types of frames from the same altitude: one with a wide-angle view (larger field of view, capturing a broad area with a smaller apparent object size) and another with a zoomed-in view (narrower field of view, focusing on a portion of the scene with a larger object scale). This simulates the effect of using a drone’s optical zoom or a camera with a different focal length while keeping altitude constant. As a result, objects of the same real-world size can appear at significantly different pixel sizes across the dataset. For example, a mid-sized car might span approximately 20 pixels in width in a wide-angle image but approximately 60 pixels in a zoomed image of the same scene. Fig. 1 (composite image of four samples) illustrates this variation: some sub-images contain dozens of small vehicles visible from afar, while others show fewer, larger vehicles close-up. This multi-scale property encourages the development of detectors that are effective across a range of object scales, similar to multi-scale training for RetinaNet or YOLO, and supports research into scale-aware or zoom-adaptive detection models in aerial imagery.

***Occlusion and Truncation***: Urban traffic scenes inherently contain occlusions, vehicles partially blocking one another, especially in dense traffic or at intersections. The UAV-OBB dataset predominantly does not include such instances because of top-down view. However, the vehicles are occluded by the trees are present in this study, we have annotated all occluded vehicle even 10% view was available, to give the better detection and tracking systems for occluded vehicles. Additionally, objects at the image edges are truncated by the frame boundary. All truncated objects are annotated to the extent visible. The presence of occlusions and truncations in UAV-OBB makes it a realistic benchmark; detectors must cope with densely packed bounding boxes and fragmented appearances, similar to real city traffic camera feeds.

***Dataset Split:*** We partitioned the data into training, validation, and test sets to facilitate standardized evaluation. Table 1 summarizes the split and class-wise distribution of object instances:

**Table 1:** Distribution of images and annotated vehicle instances across training, validation, and test splits in the UAV-OBB dataset.

Split	Images	Bike	Bus	Car	Other_Vehicle	Taxi	Truck	Total	% of Total
Train	1383	7390	3828	23,407	18,801	3328	310	40,072	85.53
Valid	218	1089	750	3523	313	498	50	6223	13.488
Test	16	126	42	282	21	33	8	512	0.99
**Total**	1617	8605	4620	27,212	2135	3859	376	**46,807**	100.00

**Table 2:** Vehicle class definitions, visual cues, and total instance count in the UAV-OBB dataset.

Class ID	Label	Includes	Visual Cues	Sample Count
0	bike	Motorcycles, scooters, e-bikes	Two-wheel profile, rider silhouette, narrow footprint	8605 (18.4%)
1	bus	City buses, coaches (>8 m)	Large rectangular body, multiple windows, roof vents	4620 (9.9%)
2	car	Private automobiles, sedans, SUVs	Compact rectangular shape, standard roof profile	27,212 (58.1%)
3	other_vehicle	Rickshaws, cargo vans, delivery vehicles, police cars	Irregular shapes, variable sizes, often three-wheeled	2135 (4.6%)
4	taxi	Marked taxis (specific color, a box on the roof)	Similar to car + roof signage, often yellow/blue in China	3859 (8.2%)
5	truck	Lorries, freight trucks, large cargo vehicles	Long rectangular body, high cabin, visible cargo bed	376 (0.8%)

As shown in Table 1, the training set contains 1383 images with a total of 40,072 annotated objects. The validation set has 218 images (6223 objects) and is intended for model hyperparameter tuning and checkpoint selection. The test set contains 16 images with 512 objects, serving as a preliminary performance sanity-check. We acknowledge that a 16-image test set is insufficient for rigorous quantitative evaluation; this is by design, as we instead provide supplementary video sequences for comprehensive evaluation. By keeping the test image set small, we encourage users of UAV-OBB to rely on the provided videos for more extensive performance evaluation in near-real-world conditions.

The class distributions indicate that car is the most frequent category (≈58% of all instances), followed by bike (≈18%), bus (≈10%), and taxi (≈8%). Other vehicles and trucks make up the remainder (≈5% and ≈1%, respectively). This distribution aligns with typical urban traffic patterns in high-density cities. Private cars dominate road usage as the primary mode of personal transportation, naturally constituting the majority of vehicles. Motorcycles and bicycles (bike category) represent the second-largest segment, reflecting their widespread adoption in many Asian cities as affordable, maneuverable alternatives for navigating congested streets and narrow alleys. Buses, while essential for public transit, operate on fixed routes with limited fleet sizes, accounting for their moderate representation. Taxis, as commercial on-demand vehicles, maintain a smaller but significant presence. The comparatively low frequency of trucks reflects urban traffic regulations that often restrict heavy goods vehicles during peak hours, while the “other vehicle” category captures miscellaneous vehicle types that appear infrequently in typical traffic scenarios.

Critically, the training and validation splits preserve these class frequency ratios with minimal deviation (car: 58.41% vs. 56.61%; bike: 18.44% vs. 17.50%; bus: 9.55% vs. 12.05%), ensuring that models trained on the dataset encounter representative examples of each class proportional to their real-world occurrence. This stratified distribution prevents class imbalance issues during training while maintaining ecological validity. Notably, although the test set is small, it contains at least a few instances of each class (ranging from 1.56% trucks to 55.08% cars) to ensure that a detector's ability to generalize to all vehicle types can be qualitatively checked. Overall, the dataset comprises 1617 images with 46,807 annotations across 6 classes, averaging 28.95 annotations per image (median: 25.0, range: 0–100).

**Fig. 2:**
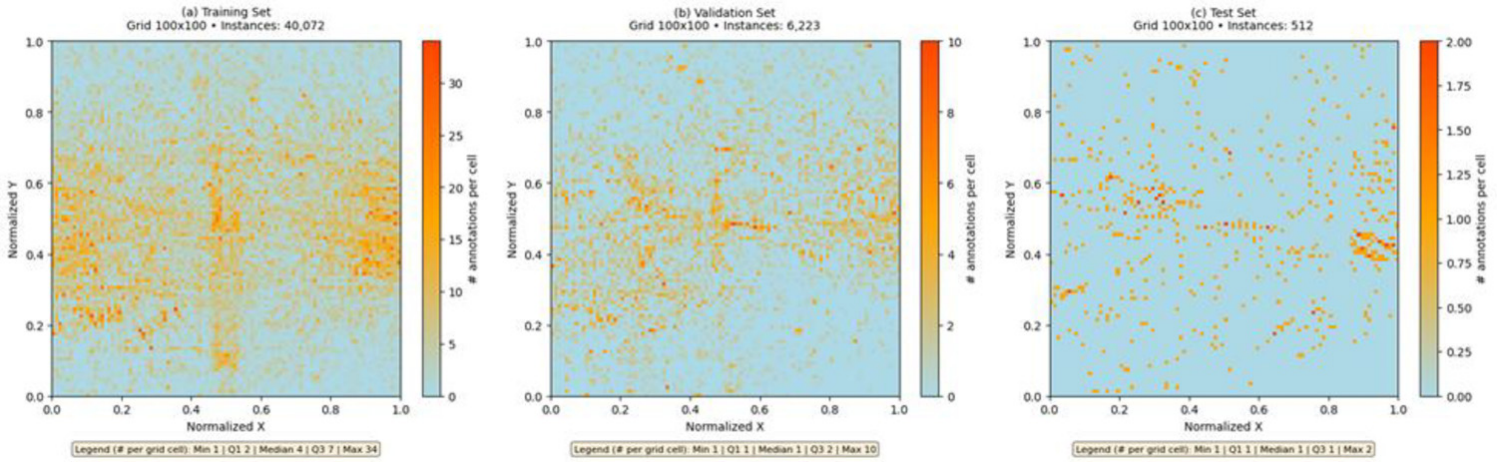
Heatmaps of vehicle center locations across training (a), validation (b), and test (c) sets on normalized 100 × 100 grids, showing realistic spatial clustering patterns with 40,072, 6223, and 512 instances respectively.

***Spatial Distribution and Realism:*** The most important aspect for any aerial dataset is whether object placements in images mimic real-world patterns (so that models trained on it will learn meaningful context). We generated heatmaps of object center locations for the training, validation, and test sets (Fig. 2) to examine spatial distribution across normalized image coordinates. These heatmaps reveal that vehicles in UAV-OBB are distributed throughout the image frame with notable clustering in certain regions, particularly visible in the training and validation sets. The training set heatmap (40,072 instances) shows moderate spatial concentration with denser regions corresponding to typical road and intersection locations captured from UAV perspectives. The validation set (6223 instances) exhibits a similar distribution pattern, confirming consistency in scene composition across splits. The test set (512 instances), while sparser due to fewer samples, maintains comparable spatial characteristics. The per-grid-cell statistics (training: median 4, Q3 7; validation: median 1, Q3 2; test: median 1, Q3 1) reflect the expected density variation given the different set sizes. These spatial distributions confirm that UAV-OBB provides realistic object placement: vehicles appear in contextually appropriate locations rather than arbitrary positions, enabling detectors to leverage spatial context such as the tendency for vehicles to cluster in traffic-dense areas. This realism in spatial layout is crucial for downstream applications such as traffic density estimation, vehicle counting, and congestion detection in urban environments, contributing to sustainable development in smart cities.

***a. Supplementary Evaluation Videos:***

In addition to the static image splits, UAV-OBB provides two video-based evaluation sets to mimic real-world monitoring where a UAV streams continuous video. Neither video is used for training, they serve purely as evaluation and demonstration data. The first is a short 60-frame clip extracted from an aerial video over a busy intersection. We manually annotated 10 frames (spaced uniformly, every 6th frame) with ground-truth oriented boxes. These serve as reference points. The remaining 50 frames have no ground-truth, but since the scene is the same, a human can readily verify whether a detection algorithm’s outputs on those frames are correct by comparing with adjacent annotated frames (hence *human-verifiable*). This novel evaluation scheme encourages algorithms that produce temporally consistent detections, a detector that accurately tracks vehicles through the unannotated frames will yield detections aligning with the eventual ground-truth frames. We envision researchers using this clip to evaluate short-term tracking performance or interpolation capabilities of their models. The second video is a 90-second continuous recording at 30 FPS (approximately 2700-frames) over an urban street. It is provided without annotations to test algorithms in a fully unsupervised setting: essentially a long stress test for detection/tracking where metrics like processing speed, memory, trajectory, and stability of detection can be observed. While no ground truth is given (preventing quantitative scoring on this sequence), it allows qualitative assessment of how a model handles prolonged operation, camera motion, and possibly changing traffic density over time. By including this video, we acknowledge that static image mAP (mean average precision) is not the only metric of interest, in practical UAV/drone surveillance, consistent performance over time is crucial. Users of UAV-OBB can run their detector or trackers on this video and inspect, for example, if false positives accumulate or if the model drifts when confronted with many consecutive frames.

***b. Comparison with Related Datasets***

Prior UAV traffic datasets such as VisDrone and UAVDT have laid important groundwork, but UAV-OBB distinguishes itself through its use of oriented boxes and its test design. VisDrone provides a large collection of drone images and videos with vehicles and pedestrians, but annotations use axis-aligned rectangles and often include many tiny pedestrian instances which require different handling. UAVDT similarly offers videos with vehicles, annotated by upright boxes and attributes like weather and camera view, but focuses on tracking in relatively simpler scenes. Our UAV-OBB, by concentrating on vehicle-centric, orientation-rich annotations, allows for more fine-grained evaluation of detection algorithms specifically for traffic surveillance. Moreover, by taking inspiration from the oriented annotation approach used in DOTA [4] (a satellite imagery dataset in 18 classes), we bring the benefits of orientation to the UAV altitude range. DOTA images are very high-altitude and include objects like airplanes, ships, and tanks, traffic data is not in dense or street view settings. In contrast, UAV-OBB objects are all common road vehicles, captured at much lower altitudes (resulting in higher object pixel sizes and more detail per vehicle). This makes our dataset particularly well-suited for training modern deep models that might otherwise struggle to learn from satellite images where vehicles are mere dozen-pixel blobs. Additionally, UAV-OBB oriented annotations can benefit and benchmark oriented-object detectors that have been developed in recent literature such as rotated RetinaNet [5], RoI Transformer [6], etc., which previously used datasets like DOTA or HRSC2016 [7] (ships) for evaluation. We acknowledge that the size of UAV-OBB is relatively smaller than the aforementioned datasets, however, its oriented annotations and real, recent urban scenes, capturing variations in time and scale, present unique and realistic conditions for researchers and planners across various fields. We expect UAV-OBB will become a valuable supplement to these datasets, enabling advancements in algorithms for aerial vehicle detection from a smart city perspective.

## 4 Experimental Design, Materials and Methods

**Fig. 3:**
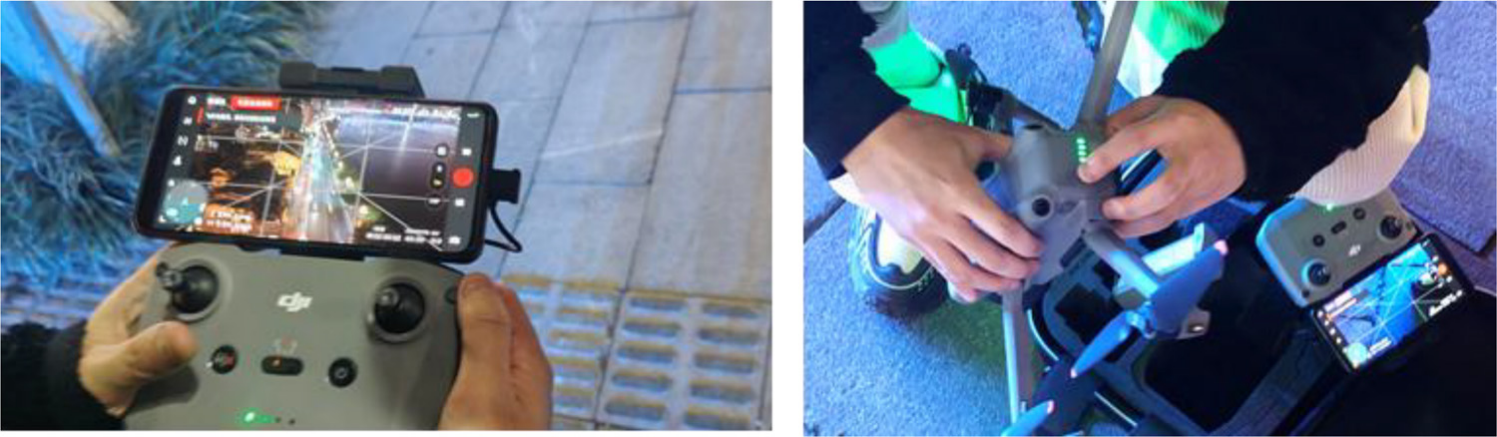
DJI Mavic 3 UAV and remote controller used for aerial data collection, showing the ground control interface with real-time video feed during urban traffic monitoring operation.

The dataset was collected using two primary methods: high-altitude fixed cameras (from tall buildings) and drones equipped with stabilized gimbals. The UAV used for data collection was a DJI Mavic 3 (as shown in Fig. 3), A compact consumer UAV equipped with a Hasselblad L2D-20c camera featuring a 4/3 CMOS sensor capable of capturing 5.1 K video at 50 fps and 4 K at 120 fps. The drone offers a maximum flight time of 46 min, omnidirectional obstacle sensing, and a transmission range of up to 15 km via O3+ technology. For this dataset, the UAV was operated in manual mode with the camera gimbal set to close to nadir view most of the time to capture top-down urban traffic scenes. Each video sequence was pre-planned to cover key traffic arteries in the target cities, Chongqing and Wuhan, capturing diverse urban layouts. All footage was captured within visual line of sight (VLOS) at altitudes ranging up to approximately 108 m Strict privacy protocols were observed; no license plates or identifiable human features were retained in the final dataset. The raw videos, recorded at 3840 × 2160 resolution and 30fps, were down-sampled to 1920 × 1080 to standardize resolution and reduce computational load.

From the video footage, image frames were sampled for annotation. Rather than using an arbitrary frame rate, we employed a content-based frame selection strategy: frames were extracted whenever the UAV was stable and the scene content changed significantly (e.g., vehicles moved substantially or new vehicles entered the frame). This resulted in a pool of candidate frames, which were manually inspected to ensure quality regarding sharpness and exposure. In total, 1617 high-quality frames were selected. The dataset was then partitioned into training, validation, and test sets. Approximately 85.53% of frames (1383 images) were assigned to training, 13.49% (218 images) to validation, and a small, representative subset of 16 images (∼0.99%) was set aside for testing. This splitting strategy ensures that no test images overlap with the training set, providing a realistic generalization test.

***Annotation Process:*** We adopted a team annotation approach. A set of two annotators were trained on how to label oriented bounding boxes using specialized annotation software we used an extension of Roboflow [8] that supports rotated boxes, as well as an in-house tool to verify YOLO OBB format compatibility. Each annotator was assigned a subset of images, and for each image, they drew a rotated rectangle around every visible vehicle. The class labels were chosen from the six categories defined, bike, bus, car, taxi, truck, and other_vehicle. We provided guidelines, such as bike includes motorcycles, scooters, and somewhere bicycles in simple terms anything two-wheeled. Truck includes trucks or lorries; other_vehicle acts as a catch-all for unusual vehicles like rickshaws, cargo vans, and delivery vans not fitting other classes. Where vehicle type was ambiguous due to distance or partial view, annotators used their judgment; in a few cases, a car vs taxi might be unclear, so the annotator checked adjacent frames or context such as taxi in China often have distinctive colors to decide.

After initial annotation, a second pass was done by a senior annotator who reviewed almost each image’s labels for consistency and completeness. Adjustments were made as needed: for example, some boxes were suggested to slightly rotate to better align with a vehicle, and some relabeling were suggested for objects where they were misclassified. We paid particular attention to occluded vehicles and included all even not leaving heavily occluded, it was still annotated to encourage detection models to learn through occlusions. The oriented nature of boxes also helped here, an occluded vehicle behind tree canopy could still get a correctly oriented box for the visible portion. On average, annotation of one image took about 08 to 14 min, given the expertise the annotators built up*.*

***Quality Control:*** To assess annotation quality quantitatively, we randomly sampled 50 images and measured the overlap between annotations from two independent annotators (each of those images was labeled by an initial annotator and then by a validator unaware of the first labels). We found that over 95% of vehicle instances had an intersection-over-union (IoU) of >0.85 between the two oriented boxes, indicating very high agreement on object extent and orientation. The few discrepancies were mostly on very small or very partially visible vehicles, which were then discussed and resolved. This gives confidence that the annotation noise in UAV-OBB is minimal.

***Data Format Details:*** The YOLOv8 OBB annotation files are plain text. Each line corresponds to one object instance and is formatted as [9], 〈class\_index〉 <*x*_1>$ $<*y*_1> 〈x_2〉 〈y_2〉 〈x_3〉 〈y_3〉 〈x_4〉 〈y_4〉, where x_i, y_i are the four corner vertices of the oriented bounding box in normalized image coordinates [0, 1] relative to the image width and height. The vertices describe the rotated rectangle as a quadrilateral (four corners). Class indices 0–5 correspond to bike, bus, car, other_vehicle, taxi, truck, respectively. For consistency, classes are enumerated alphabetically in the data files; 0 = bike, 1 = bus, 2 = car, 3 = other_vehicle, 4 = taxi, 5 = truck. An example annotation line is; 2 0.023996422213092335 0.37249611978453395 0.00025566625582035214 0.37195501993365593 −0.00032482539030399967 0.4524498615295657 0.02341593056696798 0.4529909613804437, indicates a car (class 2) whose oriented bounding box corners are located at the four normalized points (0.023996422213092335,0.37249611978453395), (0.000255-66625582035214,0.37195501993365593), (−0.00032482539030399967,0.4524498615295657), and (0.02341593056696798,0.4529909613804437). We include a script in the dataset repository to convert these OBB vertex annotations to polygon masks or axis-aligned bounding boxes when required for compatibility with alternative toolchains.

***Baseline Analysis:*** As part of the data preparation, we ran a preliminary experiment with a state-of-the-art oriented object detector on UAV-OBB to gauge its difficulty. We fine-tuned an enhanced YOLOv8 model [10] for small targets detection (with modifications for OBB) on the training set for several epochs and evaluated on validation. The overall mAP (50–95% IoU, with orientation) across all classes was 0.618, with per-class performance ranging from 0.766 for bus (highest) to 0.395 for bike (lowest). The lower performance on bikes likely reflects their small size, frequent occlusion, and sometimes indistinguishable shapes from aerial views. These results confirm that while UAV-OBB presents challenges, particularly for small or densely packed objects, it remains feasible for modern detectors to learn effectively. Notably, the model frequently predicted correct orientations, validating that angular information is learnable from the data. We emphasize that this experiment was not intended to establish a state-of-the-art detector, but rather to illustrate dataset complexity and scale variation; training was limited to 25 epochs for this diagnostic purpose. The full results are summarized in Table 3. These observations highlight areas for further research, such as improving detection in densely packed conditions, something UAV-OBB is well-suited to foster.

**Table 3:** Baseline detection performance (mAP50 and mAP50–95) per vehicle class on the UAV-OBB validation set using a fine-tuned YOLOv8-OBB model.

Class	Images	Instances	Box(P)	R	mAP50	mAP50–95
all	218	6223	0.848	0.722	0.786	0.618
bike	197	1089	0.616	0.614	0.608	0.395
bus	198	750	0.941	0.824	0.89	0.766
car	217	3523	0.876	0.857	0.893	0.736
other_vehicle	150	313	0.881	0.511	0.637	0.512
taxi	175	498	0.906	0.903	0.929	0.795
truck	48	50	0.869	0.625	0.757	0.632

***Reproducibility and Reuse:*** All raw data (images and videos) are provided in their original format except downgrading the resolution without any additional post-processing. The methods described for collection and annotation can be reproduced, for example one could extend UAV-OBB by capturing more cities using the same protocol. The dataset is formatted in a user-friendly manner, and conversion scripts are provided. This ensures that researchers can easily integrate UAV-OBB into their experimental pipelines.

***Potential Applications:*** Researchers can leverage UAV-OBB in various experimental studies. One immediate use-case is training rotated object detectors for UAV imagery, for instance, because UAV-OBB images are from real urban environments, can be used for transfer learning. One could fine-tune a model pre-trained on DOTA [11] using UAV-OBB or vice versa to possibly improve performance, an approach supported by the diversity and size of our data. Another application is in counting the vehicles inserting in a city based on their UAV-OBB training with recent urban vehicular scenes, or training a detector to detect the vehicles passing the under the trees. Moreover, the presence of sequential frames in Video A hints at multi-frame fusion possibilities, algorithms could combine temporal information to improve one-off detection. In summary, the experimental design of UAV-OBB from data collection to split, was intended to support a wide range of experimental scenarios in the context of aerial vehicle/traffic analysis in a smart city perspective.

## Limitations

UAV-OBB is designed for nadir-dominant urban traffic monitoring and its limitations are described below. First, the dataset size is moderate (1617 images) and the static test split is small (16 images), which may limit the quantitative benchmarking on the held-out test set; we mitigate this by providing supplementary videos intended for deployment-style qualitative and sparse-frame evaluation. Second, the data were collected in two cities in China (Chongqing and Wuhan), so visual appearance (road markings, vehicle fleet, taxi styles, and infrastructure) may introduce geographic bias and may require domain adaptation when transferring to other regions.

Third, class imbalance exists, such as cars are most frequent, trucks are rare, which can affect learning for under-represented categories for control setting experiments where only the performance is targeted, but this class imbalance also represents the actual urban scenario where heavy vehicles are rarely allowed, but cars are most frequent. Fourth, we will expand the sample size of the images under various weather conditions in the next phase. Finally, while occluded and truncated vehicles are annotated, heavy occlusions like under trees and very small objects remain challenging and may contain higher annotation uncertainty than fully visible instances. Despite these constraints, UAV-OBB provides consistently oriented annotations and realistic conditions that are valuable for rotation-aware detection and smart city monitoring research*.*

## Ethics Statement

The UAV-OBB dataset was collected with careful adherence to ethical guidelines and regulations. All videos were shot in public urban areas where individuals and vehicles are commonly visible from public viewpoints. The imagery primarily contains vehicles; no faces or license plate details are discernible due to the top-down angle and the altitude of capture. We have deliberately not annotated or focused on any individual person in the scenes, and any people present are effectively anonymized by distance and resolution. The data does not contain sensitive personal data.

In preparing the dataset for public release, we reviewed all images to confirm that they only depict public spaces and objects (vehicles, roads, buildings) and that no image contains any content that could be considered invasive or inappropriate. The dataset is intended for research purposes in the domains of computer vision and traffic analysis, which we believe serve the public interest, such as improving traffic safety and efficiency through better monitoring technology from a smart city and sustainability perspective. There are no human subjects’ experiments involved in this data collection beyond the operation of the UAV, and thus, institutional human subject review was not required.

Finally, the authors have no relevant ethical conflicts to disclose. We are providing UAV-OBB in the spirit of open science and with the intent that it will be used to advance technology for social good, such as reducing traffic congestion, autonomous UAV monitoring or enhancing urban planning, within appropriate ethical boundaries.

## CRediT Author Statement

**Israr Ahmad:** Conceptualization, Methodology, Data Curation, Writing Original Draft, Software. **Shang Fengjun:** Supervision, Administration, Reviewing, Validation. **Kiran Bibi:** Visualization, Software, Review and Editing. **Muhammad Salman Pathan:** Supervision, Validation, Review and Editing

## Data Availability

This dataset is released under the Creative Commons Attribution 4.0 International License (CC BY 4.0). Users are free to share and adapt the material, provided appropriate credit is given. The dataset code of our UAV-OBB annotation verification is available here. UAV-OBB (Original data) (Google Drive).

## Appendix Supplementary materials

Image, application 1

Image, application 2

Image, application 3

## Footnotes

1 Training annotations in YOLOv8-OBB.txt format; same basename as the corresponding image.

2 Ultralytics/YOLO dataset configuration file defining split paths and class names.

3 Supplementary MP4 video sequences for qualitative and sparse-frame evaluation.
